# Blood flow and coherent vortices in the normal and aneurysmatic aortas: a fluid dynamical approach to intra-luminal thrombus formation

**DOI:** 10.1098/rsif.2011.0041

**Published:** 2011-04-06

**Authors:** Jacopo Biasetti, Fazle Hussain, T. Christian Gasser

**Affiliations:** 1 Department of Solid Mechanics, School of Engineering Sciences, The Royal Institute of Technology (KTH), Stockholm, Sweden; 2 Department of Mechanical Engineering, University of Houston, Houston, TX, USA

**Keywords:** coherent vortices, intra-luminal thrombus, aortic aneurysm, platelets, computational fluid dynamics, aorta

## Abstract

Abdominal aortic aneurysms (AAAs) are frequently characterized by the development of an intra-luminal thrombus (ILT), which is known to have multiple biochemical and biomechanical implications. Development of the ILT is not well understood, and shear–stress-triggered activation of platelets could be the first step in its evolution. Vortical structures (VSs) in the flow affect platelet dynamics, which motivated the present study of a possible correlation between VS and ILT formation in AAAs. VSs educed by the *λ*_2_-method using computational fluid dynamics simulations of the backward-facing step problem, normal aorta, fusiform AAA and saccular AAA were investigated. Patient-specific luminal geometries were reconstructed from computed tomography scans, and Newtonian and Carreau–Yasuda models were used to capture salient rheological features of blood flow. Particularly in complex flow domains, results depended on the constitutive model. VSs developed all along the normal aorta, showing that a clear correlation between VSs and high wall shear stress (WSS) existed, and that VSs started to break up during late systole. In contrast, in the fusiform AAA, large VSs developed at sites of tortuous geometry and high WSS, occupying the entire lumen, and lasting over the entire cardiac cycle. Downward motion of VSs in the AAA was in the range of a few centimetres per cardiac cycle, and with a VS burst at that location, the release (from VSs) of shear-stress-activated platelets and their deposition to the wall was within the lower part of the diseased artery, i.e. where the thickest ILT layer is typically observed. In the saccular AAA, only one VS was found near the healthy portion of the aorta, while in the aneurysmatic bulge, no VSs occurred. We present a fluid-dynamics-motivated mechanism for platelet activation, convection and deposition in AAAs that has the potential of improving our current understanding of the pathophysiology of fluid-driven ILT growth.

## 1. Introduction

It is generally accepted that biomechanical conditions play a key role in the genesis and development of vascular diseases [1], and the identification of the specific causative links between biomechanics and biochemistry may help advance our current physiological and pathological understanding.

Focal enlargements of the abdominal aorta, known as abdominal aortic aneurysms (AAAs) and frequently observed in the elderly male population [2], are thought to be the end results of irreversible pathological remodelling of the arterial connective tissue [3]. The natural history of this vascular disease is frequently [4] characterized by the development of an intra-luminal thrombus (ILT) with multiple biochemical [5–8] and biomechanical [9–13] consequences. Despite ILT's impact on aneurysm disease, little is known about its development, and it is still unclear whether it increases or decreases the risk of aneurysm rupture, i.e. reinforces proteolytic activity [8], which weakens the wall [5], or buffers against wall stress [12]. It has been hypothesized that ILT develops either from rupture of vulnerable plaques (similar to thrombotic events triggered by stenotic arteries [14]) or as a more continuous process characterized by blood-flow-induced activation of platelets [15–17] and their deposition at non-endothelialized sites (or sites of endothelial dysfunction) of the wall exposed to low (sub-physiological) wall shear stress (WSS) [18].

Platelets are anucleated cells that originate from bone marrow and circulate in blood as sentinels of vascular injury. Platelet activation and adhesion can be regarded as the initiating response of thrombus formation that arrests haemorrhage in response to vascular injury and permits wound healing [19]. Pathological conditions may cause blood flow disturbances and turn this beneficial mechanism into a disease mechanism that results in aneurismatic enlargements.

Computer simulations of biomechanical phenomena are potentially significant to explore loads experienced by cells and extra-cellular components, which in turn may uncover mechanisms of mechanotransduction, i.e. how biomechanical loads are translated into biochemical signals. A simulation model represents the real object or process to a desired degree of complexity, as it is largely defined by the intended purpose. Specifically, computational fluid dynamics (CFD) simulation of blood flow remains a modelling challenge owing to complex (moving and deforming) spatial domains, constitutive nonlinearities of blood and the surrounding vascular tissues, uncertainties of boundaries and initial conditions, to mention a few.

One particularly known difficulty of CFD simulations is the analysis of the computed field variables and, at least in the biomechanical literature, simple flow visualization techniques like streamlines and contour plots have been proposed [18,20–22]. Applied to unsteady flows, such approaches yield limited understanding and even wrong interpretations of haemodynamic phenomena. In contrast, flow field coherent structures present suitable (geometrical and physical) objects to investigate complex unsteady dynamics but are seldom used for biofluid problems. The visualization of Lagrangian coherent structures [23,24] is one of the rare exceptions.

We studied unsteady blood flow in the normal and aneurysmatic aortas using Newtonian and Carreau–Yasuda constitutive models for blood, where a very suitable vortex-eduction technique, known as the *λ*_2_-method [25], was applied. Specifically, a possible connection between vortices and ILT formation was investigated, as motivated by the results from the flow over the backward-facing step (BFS) and our previous study [18]. The characterization of vortical structures (VSs) presents a powerful approach within which to describe complex flows [26–28], which overcomes the known drawbacks of Lagrangian coherent structures [29]. Despite the concept's advantages, particularly when using the *λ*_2_-method to educe vortices in unsteady flows, it has, to the authors knowledge, not yet been applied to investigate bioflows in patient-specific geometries. Most important, such an approach allows an objective explanation of physical phenomena, as it is a fundamental step in postulating a working hypothesis.

## 2. Methods

We explored the differences in flow dynamics in normal aortas and aneurysms, and tried to explain the development of ILT in AAAs. Blood flow in arteries is complex (especially in AAAs), and we approached the objective by investigating the BFS problem first, i.e. where the flow was relatively simple.

### 2.1. Geometric representation and spatial discretization

#### 2.1.1. Backward-facing step flow

**Figure 1.**
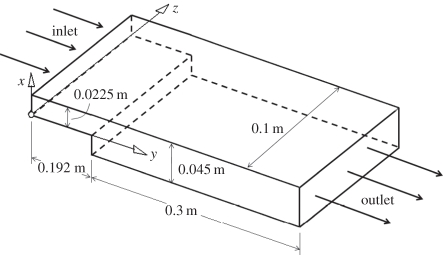
Three-dimensional geometry of the backward-facing step (BFS) problem.

The BFS flow (figure 1), a frequently applied benchmark simulation that involves flow features like separation, recirculation and reattachment, was investigated to demonstrate how recirculation bubbles are captured by the *λ*_2_-method. Note that this model flow has also been used in *in vitro* experiments (with biologically inert walls), and thrombus development is seen to start near the reattachment point of the bottom wall bubble [30].

Parameters for the BFS flow are summarized in figure 1. The two-dimensional problem used about 6000 triangular elements, and the fully three-dimensional domain was discretized by about 500 000 tetrahedral volume elements (ICEM CFD, ANSYS Inc.).

#### 2.1.2. Patient-specific normal and aneurysmatic aortas

One normal aorta, one fusiform AAA and one saccular AAA were studied in the present work, and patient-specific geometries were reconstructed from computed tomography–angiography (CT–A) scans acquired at Karolinska University Hospital, Stockholm. Standard CT–A scans (in-plane resolution: 0.6 mm; slice thickness: 1.0 mm) of the abdominal aorta were obtained with a 64-slice CT machine (Lightspeed VCT, General Electric) after intravenous injection of a bolus of iodate contrast agent (Optiray 350 mg I/ml, Tyco Healthcare). The lumen between the superior mesenteric bifurcation and 4.0 cm below the aortic bifurcation was reconstructed using active contour (deformable) models (A4research, VASCOPS GmbH). Specifically, a three-dimensionally active model, which moves under the action of external forces according to the image intensity and first and second spatial image gradients, was used to reconstruct the aortic geometry. Further details are given elsewhere [31]. It is noted that such an approach supports an artefact-insensitive [32] and automatic segmentation of the lumen, which is a fundamental requirement to provide operator-independent geometries for a reasonable haemodynamic analysis.

**Figure 2.**
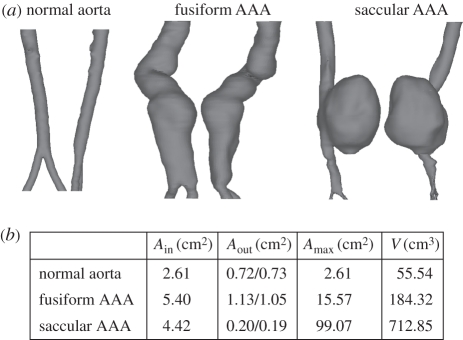
Geometries of the investigated patient-specific aortas. (*a*) Each aortic lumen is shown in two different views. (*b*) Table summarizes key geometric parameters.

The spatial domains (lumina) investigated are shown in figure 2. Specifically, the aortic cross-sectional areas at the inlet, i.e. at the site of the superior mesenteric, are given by *A*_in_, whereas *A*_out_ denotes the iliac cross-sectional area at the outlets, i.e. approximately 4.0 cm below the aortic bifurcation. *A*_max_ and *V* denote the maximum cross-sectional area and the volume of the lumen, respectively.

The Octree method [33] (ANSYS ICEM, ANSYS Inc.) was used to mesh the reconstructed spatial domains, and to facilitate a reasonable approximation of the velocity (and its gradient) in the boundary layer, and the region near the wall was discretized by prismatic elements. The remaining volume was discretized predominantly by tetrahedral elements and in total 0.6 million, 1.1 million and 0.6 million elements were used to represent the normal aorta, the fusiform AAA and the saccular AAA, respectively.

### 2.2. Mathematical model and solution procedure

#### 2.2.1. Constitutive modelling of blood

Blood has complex constitutive properties, showing strong non-Newtonian behaviour such as shear-thinning, thixotropy and viscoelasticity [34,35]. Aggregation and disaggregation of red blood cells (RBCs) is thought to determine blood's time-dependent viscosity. The aggregation process is slower, and hence defines the time scale at which blood can change its rheological properties in response to unsteady flow conditions. Among other things, RBC aggregation depends on the shear rate, haematocrit and concentrations of fibrinogen. Most interestingly, the fibrinogen concentration is elevated in AAA patients [36,37], and hence their blood might adjust faster to changes in the biochemical and biomechanical environment. Note that such experimental data are based on steady-state *in vitro* experiments, whereas unsteady haemodynamics of the vasculature enhances the mixing of blood, which in turn possibly further lowers the aggregation time.

Although it has been assumed that non-Newtonian effects may be important in complex domains [38] such as atherosclerotic regions or aneurysms [39,40] (see also early *in vitro* experiments of branching blood flow [41]), the majority of work in the biomechanical literature is still based on the Newtonian approximation, e.g. [20–23]. To investigate the impact of blood's constitutive description on the numerical predictions, two different constitutive models were considered in the present work (as detailed below) and a density of *ρ* = 1050.0 kg m^−3^ was used.

##### Newtonian model

We considered a Newtonian model with a dynamic viscosity of *μ* = 0.0044 Pa s [23].

##### Carreau–Yasuda model

To improve the Newtonian assumption and to account for the shear-thinning behaviour of blood, the Carreau–Yasuda model [42]2.1
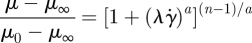
was used, where 
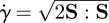
 denotes the scalar shear rate, an invariant of the rate-of-deformation tensor, i.e. the symmetric part **S** = (**l** + **l**^T^)/2 of the spatial velocity gradient **l** = grad **v** [43]. The rate-of-rotation tensor, i.e. the antisymmetric part *Ω* = (**l** − **l**^T^)/2 of **l**, as it is used (§2.3.2), was subsequently introduced.

Note that equation (2.1) describes blood as a Newtonian fluid of viscosities *μ*_0_ and *μ*_∞_ at the lower and upper ends of the shear rate range (spectrum), whereas the parameters *λ*, *n* and *a* define the transition between these extreme conditions. Constitutive parameters for the Carreau–Yasuda model, summarized in table 1, were selected to fit the experimental data of blood at 37.0°C [44,45].

**Table 1.** Carreau–Yasuda parameters to model blood's constitution at 37.0°C according to Abraham *et al*. [44] and Leuprecht & Perktold [45].

*μ*_0_ (Pa s)	*μ*_∞_ (Pa s)	*λ* (s)	*n*	*a*
0.16	0.0035	8.2	0.2128	0.64

Finally, it is noted that at a scalar shear rate of about 80.0 s^−1^, the Newtonian and Carreau–Yasuda fluids have the same viscosity. Likewise, owing to its bounded nature, the Carreau–Yasuda model has certain advantages over other non-linear models and remained still computationally highly effective (lower implementation complexity and lower computation time) by directly relating shear strain rate with shear stress.

#### 2.2.2. Backward-facing step

A homogeneous and constant velocity *V*_0_ = 0.08 m s^−1^ was prescribed at the inlet, and the no-slip condition was used at the bottom and top walls. In addition, for the three-dimensional simulation, symmetry boundary conditions at both lateral walls were used. Two-dimensional and three-dimensional steady-state solutions based on Newtonian and Carreau–Yasuda fluids were computed. The Newtonian fluid model was characterized by *Re*_*H*_ = 429 and *Re* = 859 denoting Reynolds numbers using the step height *H* = *H*_d_ − *H*_u_ and the downstream channel height *H*_u_ as characteristic length scales, respectively. The non-constant viscosity of the Carreau–Yasuda model lead to problem-dependent *Re*_H_ and *Re*, making their *a priori* calculation impossible. The considered fluid problem was entirely laminar.

#### 2.2.3. Patient-specific normal and aneurysmatic aortas

A cardiac cycle of 1.0 s was considered and the no-slip boundary condition at the wall, assumed to be rigid, was applied. A volumetric flow rate wave at the inlet and a pressure wave at the outlet (both slightly modified from a reported study [46]) were prescribed. According to this procedure the inlet velocity profile, which satisfied the no-slip boundary condition and the Navier–Stokes equations, developed during the calculation. A momentum source was added to account for the gravitational field that acts along the axial direction and simulates the upright position of the aorta. Further details regarding the applied modelling assumptions are given elsewhere [18].

A finite volume approach, using tri-linear shape functions in terms of parametric coordinates was used to solve the Navier–Stokes equations under the prescribed initial and boundary conditions (ANSYS CFX, ANSYS Inc.). Further details are given in [18].

### 2.3. Data analysis

The simulated velocity fields were imported into Tecplot (Tecplot Inc.), where smoothing was applied to reduce the numerical noise prior to further analysis. Specifically, a single Laplacian smoothing step was used, i.e. the velocity vector of a particular nodal point was redefined as the average of the velocity vectors in its first neighbourhood [47]. Finally, the spatial velocity gradient **l** was computed for the subsequent vortex eduction process (§2.3.2).

#### 2.3.1. Particle-laden flow theory

To confirm that VS presents a powerful tool with which to analyse platelet motion in blood, i.e. to understand platelet transport, we applied a particle-laden flow theory. Specifically, the Stokes number2.2
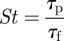
presents a dimensionless characteristic measure with which to identify how closely particles follow fluid flow, where *τ*_p_ and *τ*_f_ are particle response time and a characteristic time scale of the flow, respectively. If *St* ≫ 1, particles will continue in their own trajectories without being affected by the flow pattern. Instead if *St* ≪ 1, particles will follow the fluid streamlines/pathlines, depending on whether the flow is steady or unsteady.

The following analysis verifies that the condition *St* ≪ 1 holds for our application, and hence particle trajectories can be estimated from a macroscopic flow analysis. To this end, a conservative approach was followed, and the estimated Stokes number reflects an upper limit for the particular haemodynamic problem.

Following Yu *et al.* [48] and Fessler & Eaton [49], Reynolds number associated with a particular particle reads *Re*_p_ = *d*_p_*U*_rel_/*ν*, where *d*_p_ and *U*_rel_ denote particle diameter and the velocity characterizing the average slip velocity between flow and platelet, respectively. Considering *d*_p_ = 3.0 µm and assuming *U*_rel_ = 1.0 m s^−1^, i.e. a worst-case scenario for the slip-velocity, gives *Re*_p_ = 0.03. In contrast, the Reynolds number of the macroscopic flow problem *Re*_a_ is around 25^1^, i.e. *Re*_*a*_/*Re*_p_ ≫ 1. This allowed us to estimate the particle response time to *τ*_p_ = (2*ρ*_p_ + *ρ*_f_)*d*_p_^2^/(36*μ*) [49]. Here, *μ* is the dynamic viscosity of the fluid and *ρ*_p_ and *ρ*_f_ denote particle and fluid densities, respectively. Using the assumptions *ρ*_p_ = *ρ*_f_ = 1050 kg m^−3^ and *μ* = 0.0975 Pa s, we get an estimate for the particle response time of *τ*_p_ = 2.7 × 10^−3^ s. Taking half of the primary structures shedding (VS shedding) time *τ*_f_ = 0.5 s for the characteristic time scale of the flow, one gets *St* = 1.5*E* − 8 ≪ 1. This result assures that platelets will follow the flow [48].

#### 2.3.2. Eduction schemes

The concept of coherent structures allows for the explanation, modelling and control of complex laminar and turbulent flows. However, the underlying question ‘what actually constitutes a vortex (or VS)’ is complicated and still under discussion. VSs originate in the shear layer near the wall (also denoted as vortex sheet, a region of strong velocity gradients). This shear layer is unstable and undergoes a Kelvin–Helmholtz instability [50,51] causing a rolled-up layer of vorticity, leading to spanwise VSs. In contrast, to explain the formation of near-wall streamwise vortices (a dominant turbulence phenomenon in boundary layers) another vortex formation mechanism has been proposed [52]. Specifically, VSs are thought to be triggered by unstable low-speed streaks, where sheets of streamwise vorticity collapse (by a stretching rather than a roll-up mechanism) into streamwise vortices. VSs move, develop and interact, which involves the nonlinear processes of pairing, tearing and reconnection [27]. For a discussion of the inadequacies of common intuitive indicators of vortices such as pressure minima, closed or spiralling streamlines and pathlines, isovorticity surfaces, etc. the reader is referred to Jeong & Hussain [25].

Despite their power in analysing complex flows, the lack of an accepted mathematical definition of VSs leads to issues in their unique identification [25]. Moreover, the size of a vortex in a viscous fluid, such as blood, depends on the selected identifier's threshold.

Different eduction methods have been proposed, each of which used Galilean invariant local methods, i.e. methods based on a point-wise analysis of objective measures of the spatial velocity gradient **l**. In the present work we used the *λ*_2_-method thought to be most suitable for the underlying biofluid problem, i.e. overcoming certain limitations (drawbacks) of other approaches like the Q-method and the *Δ*-method [25].

The *λ*_2_-method assumes that a vortex structure represents a flow region surrounding a local pressure minimum [25]. It improves previously proposed searches for pressure minima by excluding contributions of unsteady straining and viscous effects. Hence, the analysis is based on a reduced form of the Navier–Stokes equation for incompressible flows2.3
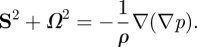


Consequently, accounting only for **S**^2^ + ***Ω***^2^ in the Navier–Stokes equation leads to an analysis of the pressure minimum solely related to vortical motion. Such a vortex core is characterized by a connected region with two negative eigenvalues of **S**^2^ + ***Ω***^2^. Since **S**^2^ + ***Ω***^2^ is symmetric, its eigenvalues are real. Ranking the eigenvalues as *λ*_1_ ≥ *λ*_2_ ≥ *λ*_3_ finally defines the equivalent condition *λ*_2_ < 0 for the presence of a VS [25]. Note that for visualization purposes, the condition *λ*_2_ = *λ*_2 tr_ is typically applied, where *λ*_2 tr_ denotes a threshold value defining the surface of the VS.

## 3. Results

### 3.1. Backward-facing step

**Figure 3.**
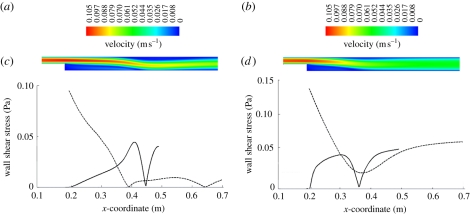
Flow field based on a two-dimensional simulation of the backward-facing step (BFS) problem using (*a*,*c*) the Newtonian and (*b*,*d*) the Carreau–Yasuda blood model. (*a*,*b*) Norm of the velocity field. (*c*,*d*) Wall shear stress (WSS) along the bottom (solid line) and top (dashed line) walls is shown.

Figure 3*a*,*b* presents a colour-coded velocity plot of the BFS from the two-dimensional simulation using Newtonian and Carreau–Yasuda blood models, respectively. While the flow fields before the step look quite similar, notable differences were present after the step; the Carreau–Yasuda model led to a more homogeneous velocity field across the channel thickness. Figure 3*c*,*d* presents the WSS distributions along the bottom and upper walls, showing a clear difference between the two models. Specifically, the Newtonian blood model predicted two recirculating regions: a larger one right after the step at the bottom wall (between *x*-coordinates 0.192 and 0.447 m) and a smaller one further downstream at the top wall (between *x*-coordinates 0.382 and 0.645 m), figure 3*c*. Interestingly, the Carreau–Yasuda blood model predicted only the large bubble (between *x*-coordinates 0.192 and 0.357 m); figure 3*d*. While the maximum WSS in the larger bubble was about the same (Newtonian model: 0.045 Pa; Carreau–Yasuda model: 0.048 Pa), its length was 35 per cent larger for the Newtonian fluid. Note also that the downstream extents of the recirculating zones depended on *Re*_H_, i.e. Reynolds number associated with the step height [53], whereas the downstream channel Reynolds numbers *Re* regulated the transition between laminar and turbulent flow. Note that the BFS flow here was laminar.

Owing to the intrinsic instability and three-dimensional behaviour of the BFS problem [54], the two-dimensional simulation enforces too restrictive constraints and the predicted three-dimensional flow field (the physically relevant one) becomes much more complicated. Naturally, the two-dimensional assumption excludes spanwise flow and hence precludes the development of streamwise VSs and prevents mass exchange between the bubble and the surrounding flow.

**Figure 4.**
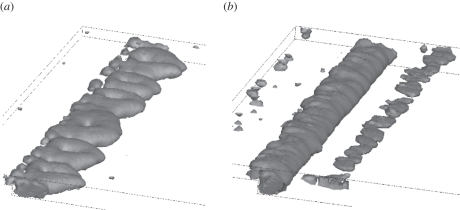
Vortical structures (VSs) of the three-dimensional simulation of the backward-facing step (BFS) using (*a*) the Newtonian and (*b*) Carreau–Yasuda blood models. VSs were educed by the *λ*_2_-method using a threshold value of *λ*_2 tr_ = −0.01 s^−2^.

A clearer picture of the complexity of flow is revealed by the *λ*_2_-method, as shown in figure 4 based on a Newtonian (figure 4*a*) and a Carreau–Yasuda (figure 4*b*) blood model where VSs were educed with a threshold value of *λ*_2 tr_ = −0.01 s^−2^. Note that the symmetry of the problem was lost in the three-dimensional simulation, especially when using a Newtonian blood model. Similar to the two-dimensional simulation, the main separation bubble was much shorter for the Carreau–Yasuda blood model. However, in contrast to figure 3*b*,*d*, this simulation showed a second (smaller) bubble at the top wall. The second bubble was suppressed with the Newtonian blood model, although it was predicted by the two-dimensional simulation, i.e. figure 3*a*,*c*. Interestingly, the (main) bubble partly covered the step (especially for the Carreau–Yasuda blood model) [54]. Finally, it is noted that the Carreau–Yasuda model predicted a spanwise VS and the Newtonian simulation showed pairs of streamwise VSs. Particularly noteworthy is the fact that the spanwise wavenumber in the Carreau–Yasuda model was twice that of the Newtonian model.

### 3.2. Patient-specific normal and aneurysmatic aortas

#### 3.2.1. Normal aorta

**Figure 5.**
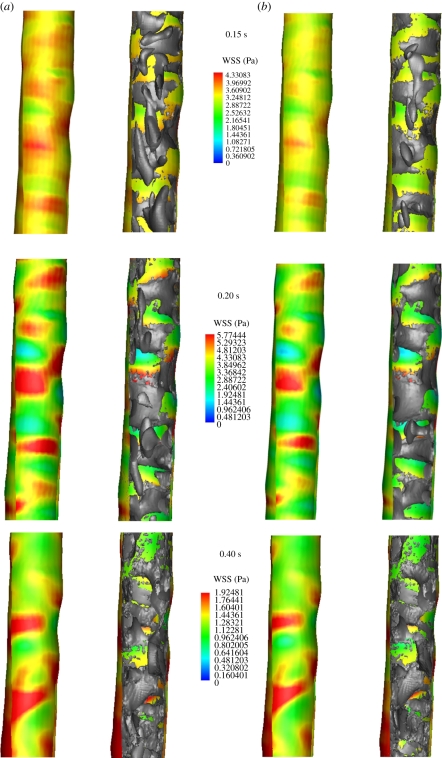
Wall shear stress (WSS) and vortical structure (VS) interaction in the normal aorta at early systole (0.15 s), peak systole (0.2 s) and early diastole (0.4 s) of the cardiac cycle. Simulations were based on (*a*) the Newtonian and (*b*) Carreau–Yasuda blood models. Luminal surface was colour-coded with the WSS and VS (educed by the *λ*_2_-method using *λ*_2 tr_ = −20.0 s^−2^) were superimposed, respectively, in grey in the right images.

At early systole (0.15 s), the flow in the normal aorta was characterized by the development of spanwise vortices all along the wall (the streamwise distance between the vortices was in the range of the arterial diameter), which predominantly formed at sites of high WSS, figure 5 (top row; 0.15 s). Sheets of spanwise vorticity rolled up into spanwise vortices, i.e. a mechanism of vortex formation suggested earlier [51], associated with the surface undulations producing local free shear layer-type profiles that then roll up by Kelvin–Helmholtz instability, as mentioned before. Once developed, spanwise vortices turn into or possibly give birth to streamwise vortices, a phenomenon predominantly observed with the Newtonian blood model. Probably, the increase in viscosity at low shear rates, i.e. in the core of blood flow, was captured by the Carreau–Yasuda model, suppressing the development of streamwise VSs. Consequently, streamwise VSs might be an artefact of the Newtonian model, which misses basic features of blood rheology. Streamwise VSs might not occur in real aortic flow fields. At peak systole (0.20 s), VSs predicted by the Newtonian and the Carreau–Yasuda model looked almost identical. Note however that compared with early systole (0.15 s), VSs remained almost unchanged for the Newtonian model (the only visible difference being the streamwise VSs that had been moved downstream), but had grown further for the Carreau–Yasuda model. Again, VS interactions with the boundary layer caused localized regions of high WSS. Owing to the higher mass flow rate entering the aorta, WSS reinforced considerably compared with early systole (0.15 s). Passing peak systole, vortex break-up was observed, i.e. VSs detached from the wall and started to disappear or break up into smaller substructures, figure 5 (bottom row; 0.4 s). Consequently, the normal aorta was free of strong (large) VSs during large parts of the cardiac cycle, see figure 7 (top row), which shows a lumen core almost free of vortexes at 0.15 s. WSS predictions between the Newtonian and the Carreau–Yasuda models were almost identical, with slightly higher values predicted by the Newtonian assumption.

#### 3.2.2. Aneurysmatic aortas

**Figure 6.**
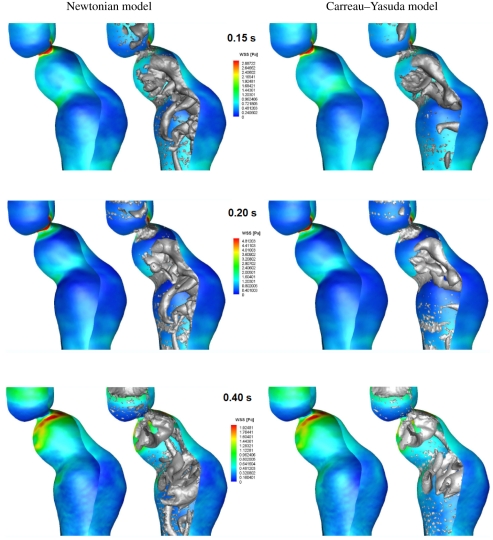
Wall shear stress (WSS) and vortical structure (VS) interaction in the fusiform abdominal aortic aneurysm (AAA) at early systole (0.15 s), peak systole (0.2 s) and early diastole (0.4 s) of the cardiac cycle. Simulations were based on (*a*) the Newtonian and (*b*) Carreau–Yasuda blood models. The luminal surface was colour-coded with the WSS and VS (educed by the *λ*_2_-method using *λ*_2 tr_ = −20.0 s^−2^) were superimposed, respectively, in grey in the right images.

**Figure 7.**
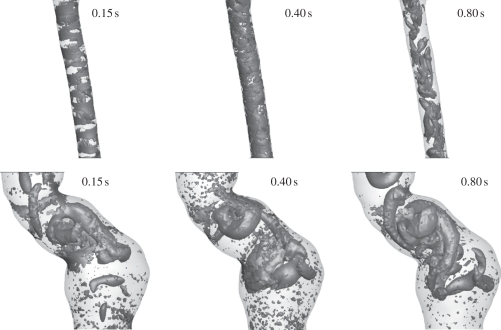
Evolution of vortical structures (VSs) in a normal aorta (top row) and in the fusiform abdominal aortic aneurysm (AAA) (bottom row) and a saccular AAA (bottom row) during the cardiac cycle. Simulations were based on the Carreau–Yasuda model and VSs were educted by the *λ*_2_-method using a threshold value of *λ*_2 tr_ = −20.0 s^−2^.

**Figure 8.**
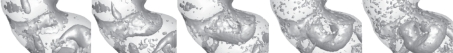
Development of a hairpin vortical structure (VS) at a strong bend of the lumen in a fusiform abdominal aortic aneurysm (AAA). The sequence starts at 0.15 s (left) and ends at 0.40 s (right) in the cardiac cycle. Simulations were based on the Carreau–Yasuda model and VSs were educed by the *λ*_2_-method using a threshold value of *λ*_2 tr_ = −20.0 s^−2^.

In the fusiform aneurysm, a few VSs developed, being much larger than those in the normal aorta. VSs strongly affected the flow, invaded large part of the lumen and were present through the entire cardiac cycle, see figures 6 and 7 (middle row). VSs were formed at sites of tortuous luminal geometry (e.g. regions of strong curvature), where pronounced shear layers and spanwise vortex sheets developed (see the evolution of VSs and their influence on the WSS in figure 6 and the different stages of a particular hairpin vortex in figure 8). A roll-up mechanism of unstable sheets of spanwise vorticity might be the main formation mechanism. Note that compared with the normal aorta, average WSS (and Reynolds number) was several times lower in the AAA [18] and shear layers suitable for VS formation were not given all along the wall but only at sites of tortuous geometry. Again, as discussed in §4, a tendency to develop streamwise VSs was observed using the Newtonian blood model. Note that owing to the strong correlation between VS impinging onto the aortic wall and WSS, even the location of the highest WSS left the neck of the AAA as soon as the VS travelled downstream (look at the location of the red spot between peak (0.2 s) and late (0.4 s) systole in figure 6).

In the saccular AAA, the strong curvature at the beginning of the aneurysmatic portion triggered a spanwise horseshoe VS, which moved downstream and tears into two worm-like streamwise vortices that finally entered the distal portion of the aorta, figure 7 (bottom row). Owing to low flow velocities (i.e. low Reynolds number) and low shear rates, the aneurysmatic bulge was free of VSs throughout the entire cardiac cycle. Finally, it was noted that the saccular aneurysm was only analysed with the Carreau–Yasuda model since the Newtonian model could clearly not account for the higher blood viscosity in the aneurysmatic bulge, i.e. where blood was almost at rest.

In contrast to the normal aorta, where VSs started to disappear or break up into smaller substructures after peak systole (0.2 s), no such phenomenon was seen in the AAA. On the contrary, it seems that VSs (particularly streamwise VSs) even reinforced towards late diastole (0.8 s), figure 7.

As in the normal aorta case, WSS predictions in the fusiform AAA between the Newtonian and the Carreau–Yasuda models were similar, although VSs differed significantly, figure 6. While in the normal aorta, only at early systole (0.15 s) was a clear difference between the Newtonian and the Carreau–Yasuda models observed; in the AAA, the Newtonian model predicted more VSs at any time, figure 6. Specifically in the core flow, the lower shear rate 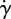 using the Carreau–Yasuda model led to a higher blood viscosity, which in turn leads to less VSs.

**Figure 9.**
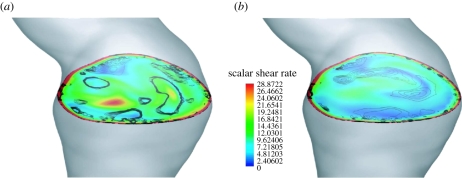
Local differences of the results as predicted by (*a*) the Newtonian and (*b*) the Carreau–Yasuda model. Images show the colour-coded scalar shear rate 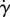 at a selected plane in the abdominal aortic aneurysm (AAA) at peak systole (0.2 s) overlaid by the contours of vortical structures (VSs). VSs were educted with the *λ*_2_-method using threshold values of −20.0 s^−2^ ≤ *λ*_2 tr_ ≤ 0.

To quantify the (qualitative) observation, we introduced the *L*_2_ norm of the vorticity within the domain *V* according to2.4
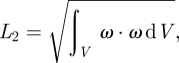
where *ω* denoted the vorticity vector, i.e. *ω*_*i*_ = −*ɛ*_*ijk*_*Ω*_*jk*_, with the alternating symbol *ɛ*_*ijk*_. Note that the *L*_2_ norm captures vorticity regardless of whether it arises from a VS or from the boundary layer. Results from that analysis are summarized in table 2, which underlines that the Newtonian model predicted up to 10.6 per cent more vorticity when compared with the Carreau–Yasuda model. This integral measure, i.e. the *L*_2_ norm, can naturally not quantify local differences, as they become apparent when investigating the scalar shear rate 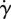 for example. Figure 9 clearly illustrates the difference in 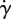 at a selective plane in the AAA at peak systole (0.2 s), i.e. the time in the cardiac cycle, where the *L*_2_ norm only differed by 3.8 per cent between the Newtonian and Carreau–Yasuda models. Note that the selected cross section in figure 9 had a large diameter, which led to a (globally) small shear rate and defines a (globally) high viscosity of the Carreau–Yasuda model. Consequently, plotting a shear stress-related parameter instead of the shear rate in figure 9 would show less global difference between the Newtonian and Carreau–Yasuda fluids.

**Table 2.** *L*_2_ norm comparison between predictions based on the Newtonian and the Carreau–Yasuda models of the fusiform AAA.

time in cardiac cycle (s)	*L*_2Newtonian_	*L*_2Carreau-Yasuda_	relative difference (%)
0.15	0.225	0.204	9.3
0.20	0.364	0.350	3.8
0.40	0.262	0.242	7.6
0.80	0.170	0.152	10.6

## 4. Discussion

Haemodynamics is thought to play a key role in how biomechanical loads are translated into biochemical signals. However, results from CFD simulations are not always easy to interpret. Vortices are the muscle and the voice of a flow, being the workhorses of transport phenomena and noise production, particularly in turbulent and vortex-dominated flows [27]. Here, we employed VSs as an objective characterization of physical phenomena in blood flow in larger arteries towards postulating a working hypothesis regarding the initiation and development of ILT in AAAs.

**Figure 10.**
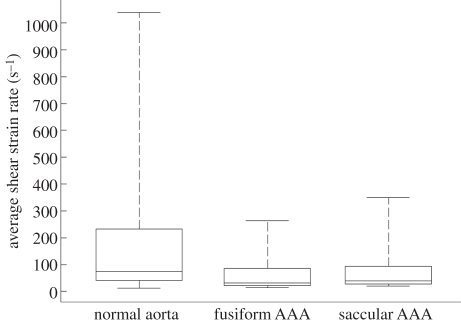
Distribution of the scalar shear rate 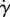 in the normal aorta, fusiform AAA and saccular AAA represented by box (lower and upper quartiles) and whisker (minimum and maximum) plots. Data were collected from the last cardiac cycle (200 data points) and averaged over the entire simulation domain. The normal aorta showed the highest maximum value, corresponding to peak systole, owing to the restricted lumen with respect to the other cases. The saccular AAA showed a higher maximum compared with the fusiform AAA owing to the smaller outlet sections and to the influence of the aortic section resembling a healthy vessel, figure 2.

Different eduction schemas for VSs have been proposed in the literature, and the present study was based on the *λ*_2_-method [25]. In this perspective, capturing a vortex meant an identification of low-pressure regions, within which the flow was characterized by vorticity, whereas shear dominated at their periphery and further outside. Apart from investigating flow features like vortex advection, distortion, break-up and vorticity diffusion (which by themselves gave a detailed picture of the haemodynamic events), the low Stokes number of our problems implied that platelets closely followed the flow, and hence vortex motion was effective in assessing platelet motion. Naturally, such an analysis required a reliable prediction of VSs, which however was found to be largely dependent on the underlying constitutive model for blood. While the near-wall flow was not affected by the viscosity model of blood, the Newtonian model predicted VSs in the core flow, which did not appear when using the Carreau–Yasuda model, thought to capture blood rheology more accurately. Consequently, the Newtonian model seems to be inadequate, in particular for diseased states, where the increase in viscosity in the core flow at large vessel diameters (i.e. at low shear rates) cannot be captured. It is noted that particularly for the aneurysmatic aorta, i.e. which shows globally a lower shear rate (figure 10) than the normal aorta, a higher viscosity compared with the one of the Newtonian model than it is generally used in the literature (i.e. *μ* = 0.0044 Pa s) might be justified. Finally, studying the impact of more complex rheological models (taking into account thixotropy for example) on the flow field was beyond the scope of the present work, but is worth investigating in future studies.

Platelet adhesion and aggregation can be regarded as the initiating mechanism of thrombus formation, and high shear stress in blood flow (a condition present at the periphery of the VS, for example) may lead to purely von Willebrand factor (VWF)-mediated platelet aggregation, i.e. without the presence of agonists, see [19] and references therein. Specifically, the integral of shear stress over time, i.e. ∫*τ* d*t* [15], has been proposed as a possible (biomechanical) activation parameter. While continuous release of antagonists from the endothelial layer prevents platelet activation and aggregation close to the vascular wall, i.e. at the site of highest shear stress under normal haemodynamics, this mechanism seems to be altered in diseased vessels.

**Figure 11.**
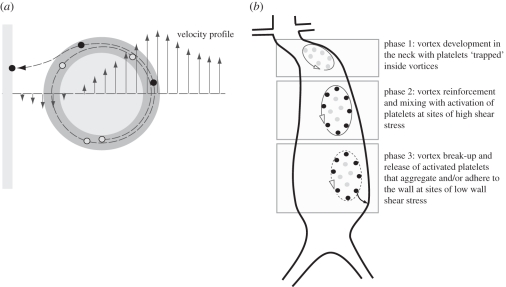
Schematic of the suggested mechanism of intra-luminal thrombus (ILT) formation in abdominal aortic aneurysms (AAAs). (*a*) A platelet travels at the boundary of a vortex (i.e. in the region of high shear stress) prior to it being released and attaching itself to the wall at sites of low shear stress. Light grey, low shear stress region; dark grey, high shear stress region. (*b*) Different phases of ILT formation in an AAA. Phase 1: vortices are formed in the geometrically complex neck region and they capture non-activated platelets (grey dots). Phase 2: vortices reinforce and become mixed up while travelling downstream. Residence times are in the order of seconds and high shearing at the periphery of the vortices activates platelets (black dots). Phase 3: the vortex breaks up in the distal part of the AAA and releases activated platelets, which in turn can aggregate and/or attach to the wall.

AAAs are usually defined by a tortuous neck region forming strong VSs, which last over the entire cardiac cycle, see the example investigated in §3.2. Consequently, platelets may be exposed to much larger values of ∫*τ* d*t* than in the normal aorta, i.e. where no such strong VSs could be identified. A high value of ∫*τ* d*t* could either be caused by the long residence time inside the vortex, or owing to the high shear stresses on the periphery of the vortex. Here, a ‘strong VS’ is defined as a vortex with a large circulation, i.e. a large area integral *Γ* = ∫*ω* d*A* of the vorticity *ω*. The proposed ILT formation mechanism in AAAs is illustrated in figure 11.

A vortex can also be seen as an island [55], i.e. shielded from the surrounding flow and that can hardly be penetrated by antagonists, which otherwise would prevent platelet activation. Note that an intact endothelial layer, i.e. one able to release antagonists, is anyway seldom observed in larger (clinical relevant) AAAs.

The results of the present study also showed that the downward motion of vortices in the fusiform AAA was in the range of a few centimetres per cardiac cycle, such that release (from a vortex) of activated platelets may still be within the lower part of the aneurysmatic bulge (figure 11), i.e. where the thickest ILT layer is frequently observed. Specifically, an analysis of non-ruptured AAAs (*n* = 30) (details regarding the patient material is given elsewhere [56]) showed that ILT starts below the neck and becomes thickest in the lower part of the aneurysm in two-thirds of the cases. Some of the other cases did not show geometrical tortuosities strong enough to trigger strong vortices. In contrast, an ILT is seldom observed in saccular AAAs, and the absence of VSs in the aneurysmatic bulge, as predicted by the present study, could explain that. Note that VSs in the saccular AAA were close to the healthy portion of the aorta, where an intact endothelial layer would release antagonists and hence, prevent ILT formation. Note that a detailed study regarding the impact of the AAA neck geometry on the VS was beyond the scope of this work, although its outcome might be of the highest (clinical) interest.

Note that activated platelets could adhere to the non-endothelialized (thrombogenic) surface, where the low WSS (as typically seen in AAAs [18]) promotes adherence to immobilized substrates like VWF, collagen, fibronectin, fibrin, thrombospondin and laminin [19], most of them being found in the ILT. Probably, the adherence rate is highest at the site of lowest WSS, as experimentally indicated by the BFS problem, where thrombus formation was first identified in the vicinity of the reattachment point at the bottom of the channel, see [30] and §3.1. Once platelets adhere, maturing of ILT tissue is defined by the development of a fibrin-based tissue, which later turns, via a granulous-type tissue, into a fibrillar collagenous tissue [57].

Finally, it is emphasized that different pathways of platelet adhesion are activated at different shear stress levels and that platelets bind through a variety of specific membrane receptors to cells and extracellular matrix constituents, see [19] and references therein. While our assumption, i.e. that the thrombus grows at sites of low WSS, will prevent luminal occlusions, as they are otherwise typical for thrombotic events following plaque rupture. In this latter case, an intact endothelial layer might support an adhesion mechanism activated at high WSS, between the VWF A1 domain and the glycoprotein VI receptor, for example.

In conclusion, VSs allow a more detailed assessment of salient haemodynamic conditions of the normal and diseased aorta, as is required in order to explore vascular malfunctions. Apart from *in vitro* experimental work towards thrombus formation under defined biomechanical and biochemical boundary conditions, a particle (platelet) tracking analysis might be particularly helpful to quantify some of the statements and questions raised by this work. In this respect, the integration of biochemistry, i.e. implementing platelet activation pathways on top of the flow-field equations, is most important.

## Footnotes

1 Calculation based on the fusiform AAA.
